# Emotions and Instructed Language Learning: Proposing a Second Language Emotions and Positive Psychology Model

**DOI:** 10.3389/fpsyg.2020.02142

**Published:** 2020-08-25

**Authors:** Kaiqi Shao, Laura J. Nicholson, Gulsah Kutuk, Fei Lei

**Affiliations:** ^1^School of Foreign Languages, Hangzhou Dianzi University, Hangzhou, China; ^2^Faculty of Education, Edge Hill University, Ormskirk, United Kingdom; ^3^Faculty of Education, Edge Hill University, Ormskirk, United Kingdom; ^4^School of Foreign Studies, Center for Language Cognition and Assessment, South China Normal University, Guangdong, China

**Keywords:** emotion, second language acquisition, positive psychology, theory, learning, teaching

## Abstract

Although emotion research and positive psychology (PP) have recently gained strong momentum in the field of second language acquisition (SLA), theoretical models linking language emotion and PP research, which offer insights for both research and intervention practice are lacking. To address this gap, the present article first introduces the origin, concept, and research around PP. Next, it summarizes recent research on PP and emotions in SLA. Finally, by triangulating emotion theories and research in the fields of psychology, education, and SLA, we propose a new model, which merges the three pillars of PP (positive institutions, positive characteristics, and positive emotions) with the antecedents, outcomes, and interventions of second language (L2) emotions (the L2EPP model). The value of the model to L2 pedagogy and research is highlighted in the context of the importance of integrating PP into the area of emotions and instructed SLA.

## Introduction

Language learning creates a spectrum of emotions of both positive and negative connotation. These emotions are of critical importance for second language (L2) learning and achievement (Shao et al., 2019). However, past research on emotions and second language acquisition (SLA) has traditionally focused on negative emotions, particularly language anxiety (see Teimouri et al., 2019, for review), leaving the effects of positive emotions (e.g., happiness, pride, gratitude, joy, hope, and admiration) largely unaddressed (Dewaele and MacIntyre, 2014). Recently, there has been a move toward examining a few positive emotions (e.g., enjoyment; Dewaele et al., 2018), as well as the positive effects of negative emotions (Swain, 2013) in L2 teaching and learning. Examining different language learning emotions together with their antecedents and outcomes from a more positive rather than solely negative perspective permits a balanced insight into how language learners are able to fine-tune their emotions that eventually leads them to success in the long run of SLA (Oxford, 2016).

According to Gable and Haidt (2005), positive psychology (PP) can be defined as the scientific study that aims to better understand the conditions and processes involved in the flourishing or optimal functioning of groups and individuals. Seligman and Csikszentmihalyi (2000) pointed out that PP research is built on three pillars: positive emotions (e.g., happiness and joy), positive characteristics (e.g., strengths and virtues), and positive institutions (e.g., family and school). As a key component of PP, positive emotions are seen not only as end states of flourishing and happiness, but also as a means to achieving psychological growth, intellectual development, and improved well-being over time (Fredrickson, 2001). This suggests that they are worth cultivating and may play a central role in SLA, which is a gradually developing process necessitating long-term effort, motivation, interest, resilience, optimism, and the like (MacIntyre et al., 2019). Moreover, positive characteristics such as empathy, courage, optimism, and trait emotional intelligence, which are reflected in thoughts, feelings, and behaviors, can energize the language learners, helping them to recognize their own and others’ strengths, overcome language obstacles, and obtain optimal affective and learning experience in the L2 classroom (Lake, 2013; Shao et al., 2013). Good institutions/schools, which are characterized by security, democracy, freedom of inquiry, high quality of education, and teacher and peer support can transcend the lone individual L2 learner, strengthen positive characteristics, and engender positive emotions to better a community of language learners (Peterson, 2006; Khajavy et al., 2018). These three pillars of PP are intricately linked with one another in the process of SLA as an individual L2 learner’s positive characteristics can generate positive emotions, which can have a ripple effect on peers and teachers through mechanisms such as emotion contagion and social appraisal (Parkinson, 2019), and thus influence the emotional climate at classroom and institutional levels, resulting in a beneficial effective experience and language development for all (Khajavy et al., 2018).

Riding on the wave of PP in SLA, these core concepts of PP may serve as a useful guide for L2 emotion researchers to expand the scope of emotions investigated, examine the causes and consequences of multiple emotions in more depth, and provide concrete recommendations for L2 teachers to foster students’ beneficial emotional experiences (Dewaele et al., 2019a; Shao et al., 2019). The present article aims to contribute to our knowledge and understanding of these important topics by first briefly synthesizing existing literature on PP and emotions in SLA, and then introducing a theoretical model, namely, L2EPP (second language emotions and positive psychology), which integrates emotion theories and research in the fields of psychology, education, and language with preliminary PP intervention studies in SLA. We anticipate that the interdisciplinary nature of this model will promote more research opportunities for L2 researchers, as well as the practical improvement of instruction in language classes.

## An Overview of Positive Psychology

PP has become a buzzword in the field of psychology in the past two decades. The historical basis of this concept can be traced back to the teaching of ancient Greek philosopher Aristotle, who emphasized the importance of happiness and well-being in life, which he referred to as *eudaimonia* (Robinson, 1991). With its modern roots in the humanistic approach of psychology (Maslow, 1954), PP, as a structured discipline, was first introduced in Martin Seligman’s 1988 Presidential Address of the American Psychological Association (Seligman, 2002; Gable and Haidt, 2005). Initially, the basic premise of PP was that human beings are bestowed with positive traits and driven by the passion to seek a happy, engaged, and meaningful life. It was envisioned that psychology needed a more optimistic outlook by studying topics such as character strengths, love, happiness, well-being, and wisdom rather than being preoccupied by the abnormal and illness mentalities (Seligman and Csikszentmihalyi, 2000). PP did not aim to disregard negative emotions and psychological disorders but was proposed as a complementary approach that could help to balance pathology-focused approaches, which might produce only limited understanding of human nature (Peterson, 2006).

The concept of PP at the subjective level is about positive subjective experience: well-being and satisfaction, flow, joy, the sensual pleasures, and happiness; and constructive cognitions about the future – optimism, hope, and faith. At the individual level, it is about positive personal traits: the capacity for love and vocation, courage, interpersonal skill, aesthetic sensibility, perseverance, forgiveness, originality, future-mindedness, high talent, and wisdom. At the group level, it is about the civic virtues and the institutions that move individuals toward better citizenship: responsibility, nurturance, altruism, civility, moderation, tolerance, and work ethic (Seligman and Csikszentmihalyi, 2000). As the three pillars of PP, positive emotions, positive characteristics, and positive institutions are tied to the scientific inquiries of what makes life worth living and what helps people flourish in life (Peterson, 2006).

PP is a science that emphasizes theory-practice integration. Since Seligman and colleagues co-initiated the special issue of PP in the journal of *American Psychologist* (Peterson, 2000; Seligman and Csikszentmihalyi, 2000), a large number of empirical research has been conducted to attest to the theoretical soundness of PP constructs. In general, findings provided support for the tenets of PP, showing that each pillar of PP is pivotal for human beings’ cognitive and affective functioning, coping and resilience to adversity, physical and psychological health, and increased engagement, performance, and satisfaction with their work and life (e.g., Fredrickson and Branigan, 2005; Rode, 2013; Meneghel et al., 2016). Seligman et al. (2005) also developed and refined a list of 40 practical interventions for practitioners to use to help people increase happiness. For instance, some of the well-known interventions include: expressing gratitude to an important person, describing three good things that happened each day, identifying one’s signature strengths, and using signature strengths in new ways. Two meta-analyses, which included 49 and 39 studies, respectively, supported the effectiveness of PP interventions for improving well-being and lowering depression, and the positive effects of these interventions on psychological well-being remained significant after 6 months (Sin and Lyubomirsky, 2009; Bolier et al., 2013).

Theory and practice of PP have evolved in recent years. The focus of PP has shifted from happiness (positive emotions, engagement, and meaning) to a more well-rounded theory of well-being. In his book, “Flourishing”, Seligman (2011) explicitly states that “…the topic of positive psychology is well-being, that the gold standard for measuring well-being is flourishing, and that the goal of positive psychology is to increase flourishing” (Seligman, 2011, p. 13). According to Seligman (2011), human flourishing can only be achieved through enhancing each and every aspect of PERMA: positive emotion, engagement, meaning, positive relationships, and accomplishment. Individuals in such a state thrive, feel vitality, and prosper at both individual and institutional levels. Furthermore, the term “second wave positive psychology” represents a call in the field of PP to integrate conceptualization of positive and negative processes and to understand the role of contexts to create more appropriate theoretical accounts about psychology in practice (Lomas and Ivtzan, 2016). Combining the study of positive and negative experiences allows theory and research to move beyond simple, static, and linear descriptions of emotions, attitudes, and cultures (Snyder et al., 2015). These new developments of PP coincide with the introduction and flowering of PP in emotions and SLA.

## PP and Emotions in SLA

### PP Constructs in SLA

Although the explicit introduction of PP into the field of SLA has only been a recent phenomenon, earlier studies on the “good language learner” (e.g., Naiman, 1978) as well as L2 motivation research such as Gardner’s (1985) socio-educational model and Dörnyei’s (2005) L2 self-system model have all touched on the positive aspects of language learners and demonstrated the importance of positive characteristics, positive attitudes, and positive emotions for the successful acquisition of a second language.

MacIntyre and colleagues (MacIntyre and Gregersen, 2012; MacIntyre and Mercer, 2014) were the first to formally bring PP into the spotlight of L2 researchers. They referred to Fredrickson’s broaden-and-build theory (BBT) of positive emotions and its associated practical functions (see MacIntyre and Gregersen, 2012, for a summary) and argued that teachers have the potential to help students harness both positive and negative emotions by using techniques such as promoting imagination and practicing relaxation in language classrooms. Their work highlighted the importance of PP theories for language teaching, learning, and communication, and identified promising trends such as the move toward studying positive emotional states (e.g., love, enjoyment, and flow) and learner strengths (e.g., courage, empathy, and hardiness) in SLA. They also addressed and proposed solutions for criticisms of PP that also occurred in L2 contexts (e.g., measurement issues and over-reliance on cross-sectional data; MacIntyre et al., 2016, 2019).

In another body of work, Oxford (2016) expanded Seligman’s (2011) PERMA framework by drawing upon prior research in psychology and proposing a more encompassing model containing key elements that promote happiness within language learners and teachers. Specifically, the model comprises emotion and empathy; meaning and motivation; perseverance (including hope, resilience, and optimism); agency and autonomy; time; hardiness and habits of mind; intelligence; character strengths; and self-concept, self-efficacy, self-esteem, and self-verification, and is known using the acronym, EMPATHICS. Oxford (2016) put forth a series of testable hypotheses related to each dimension of the model, and the preliminary empirical findings appeared to support the efficacy of the classroom interventions. Lake (2013, 2016) and Mercer (2016), to name a few, also investigated a small number of constructs in this framework, such as courage, self-concept, and empathy and their initial findings provided support for the usefulness of these variables for L2 teaching and learning at both individual (in terms of enhancing positive L2 self, L2 self-efficacy, and L2 intended effort) and institutional level (in terms of promoting social relationships, positive group dynamics, and optimal classroom atmosphere).

Dewaele and colleagues have also emphasized the importance of adapting a more holistic view of L2 learners’ emotions by focusing on both negative and positive emotions (Dewaele and MacIntyre, 2014; Dewaele et al., 2018). They proposed that positive emotions promote students’ resilience and perseverance to overcome language difficulties and encourage learners to explore and play, which are crucial for building social cohesion (Dewaele et al., 2018). Their work on foreign language enjoyment (FLE) has shifted L2 researchers’ attention from their previous preoccupation with negative constructs, such as anxiety, to positive emotion constructs, which marks a new era of investigation. This research has triggered a series of recent studies examining antecedents and outcomes of FLE in different contexts (to be discussed next; e.g., Jin and Zhang, 2018; Saito et al., 2018; Dewaele et al., 2019b; Li, 2020).

### Positive Emotions in SLA

Before the advent of PP in SLA, research in the field of emotions and language learning has been dominated by anxiety, which is the only emotion that has been systematically investigated (MacIntyre, 2017; Teimouri et al., 2019). However, even in this “negative” period, one could add that signs of PP were already present in studies that investigated the effects of positive characteristics such as trait emotional intelligence on language anxiety (Dewaele et al., 2008; Shao et al., 2013). The role of positive affect has also featured in more teacher-oriented research by Arnold (1999) since the turn of the century. Theoretical and empirical introduction of positive emotions in SLA started from the work of MacIntyre (MacIntyre and Gregersen, 2012) and Dewaele (Dewaele and MacIntyre, 2014). Following the BBT (Fredrickson, 2001), they pointed out the beneficial functions of positive emotions for language learners in terms of broadening cognition, tempering negative emotions, promoting resilience, building personal and social resources, and triggering a virtuous circle toward greater well-being and achievement (MacIntyre and Gregersen, 2012; Dewaele et al., 2019a). Employing these concepts, Dewaele and MacIntyre (2014) explored one type of positive emotions: enjoyment, among 1,746 foreign language (FL) learners from around the world. Findings revealed that participants reported higher levels of FLE than foreign language anxiety (FLA), and FLE was only moderately negatively correlated with FLA. FLE and FLA were also linked with socio-demographical variables such as gender, age, number of FL known, and perceived language proficiency. Qualitative data indicated that teachers’ professional and emotional skills and peer support are important factors influencing learners’ FLE.

Inspired by this pioneering study, L2 researchers began to investigate a wide range of predictors (internal vs. external) and outcomes of FLE as well as the dynamic interactions among these variables from a PP perspective. For example, Dewaele et al. (2018) examined the extent to which certain learner and teacher variables were linked to students’ FLE and FLA. Results from 189 British high school students learning various foreign languages in class showed that FLE was negatively correlated with FLA but positively related to achievement. Students’ age, gender, degree of multilingualism, language proficiency, and attitude toward the foreign language were also related to their FLE. Interestingly, teacher variables such as unpredictability of the class, frequency of L2 use, and students’ attitudes toward the teacher were more strongly correlated with students’ FLE than to their FLA. Moreover, employing a mixed cross-sectional and longitudinal design, Saito et al. (2018) investigated how language learners’ emotion and motivation profiles related to their oral proficiency among 108 Japanese high school students. Results showed that learners’ enjoyment and motivation, but not anxiety, were positively related to studying, practicing, and using the target language throughout their L2 learning experience. Students with a clear vision of their ideal L2-Self experienced more enjoyment and less anxiety in their language learning. Students’ enjoyment also positively predicted both their long-term and short-term language achievement, while anxiety only influenced their long-term achievement. In the Chinese context, Jin and Zhang (2018) investigated factors underlying FLE among 320 EFL high school students. Factor analysis yielded a three-factor solution from an adapted FLE scale: enjoyment of teacher support, enjoyment of student support, and enjoyment of FL learning. FLE exerted both direct and indirect effects on students’ FL performance. Enjoyment of FL learning had the strongest effect on achievement scores with enjoyment of teacher support and enjoyment of student support having indirect effects.

Taken together, this initial evidence highlights the importance of positive emotions in enhancing L2 learners’ motivation and performance and decreasing their language anxiety in the long run and suggests that L2 teachers should strive to boost students’ enjoyment rather than solely focusing on reducing their anxiety in language classrooms (Dewaele et al., 2018). However, it is noteworthy that these initial studies probed only one type of positive emotions; enjoyment, but other pleasant emotions such as hope, pride, contentment, gratitude, and admiration may play an equally important role in promoting L2 learners’ motivation, creativity, interest, and performance and thus are also worth investigating. Recent research has started to address this by adapting the construct of achievement emotions from the field of educational psychology to the FL context in order to measure a wider range of positive emotions: enjoyment, hope, and pride (Shao et al., 2019, 2020). This line of research will be discussed in more detail later on.

### A More Balanced Approach to the Study of Emotions in SLA

Recent hermeneutics on PP emphasize the need to integrate positive and negative experiences and move beyond the static notion of seeing them as simply good or bad (Snyder et al., 2015). As Prior (2019, p. 522) pointed out, “Any emotion can be facilitative or restrictive, motivating or demotivating, adaptive or maladaptive. To fully engage with emotion in language research and teaching requires a focus on context and a willingness to simultaneously embrace ‘joy’ as well as ‘pain’.” First and foremost, this foregrounds a more balanced meta-theoretical perspective on the influence of negative emotions in the language learning process, rather than treating them as purely maladaptive. Indeed, some research has demonstrated the “positive power” of negative emotions for L2 learners to acquire the target languages.

For example, Swain’s (2013) study showed that learners’ embarrassment may serve as a strong impetus for them to acquire the L2 in order to avoid such feelings in front of peers; while anger can motivate some students to master the second language as a way of revenging on perceived unfair treatment by the school principal. Similarly, López and Cárdenas (2014) found that Mexican students were able to transform negative emotions such as fear, anger, and frustration toward teachers’ written feedback and evaluations into positive energy after reflecting on their moral obligations as students to their families. Cultural value on education, socio-economic condition, family issues, and aspiration are documented as mediators in the relation between teacher feedback and students’ emotions and writing texts. In the Japanese context, Imai (2010) examined how a group of EFL learners discursively constructed and shared their emotional attitudes toward a semester-long group assignment that pushed them to co-construct their knowledge and challenge the assigned tasks and material. Participants’ narratives show that emotions like boredom, discontentment, and frustration could become a psychological resource for their L2 development and mediate the relationship between cognitive task demands and subsequent learning behavior.

Importantly, these studies imply that negative emotions are seemingly detrimental and may initially lower students’ motivation; however, they may be turned into motivational fuels, depending on how L2 learners can make sense of these emotions and manage their emotionality to their advantage rather than succumbing to it. This meaning-making process is in line with the essence of PP, which emphasizes the importance of learning lessons from negative experiences and finding positive meaning in the face of adversities (Peterson, 2006). Such complex effects of negative emotions are more readily observed from anxiety research in education and language showing that anxiety can have either positive, negative, or zero correlations with performance (e.g., Kleinmann, 1977; Eysenck et al., 2007; Marcos-Llinás and Garau, 2009). Furthermore, these findings demonstrate that negative emotions and positive emotions often co-exist in the language acquisition process and may interact with cognition and contextual factors in affecting language learning and use. For example, family responsibility, economic condition, and collective culture were important contextual factors that shaped the emotions of enjoyment, pride, and hope in the studies by Imai (2010) and López and Cárdenas (2014). The interplay among emotion, cognition, and context may take a more complex non-linear form than any experimental or cross-sectional research has assumed. Rather than denying the positive side of negative emotions, it may be best to find a leverage point on the continuum of positive emotions and negative emotions with a consideration of the language learning context.

Further, it is important to note that the effects of positive emotions on language learning are also context-dependent and may not always be adaptive (Komorowska, 2016). From the PP perspective, this implies a more balanced approach regarding the influence of positive emotions in SLA as well. Research in psychology and education has shown that positive emotions, under certain circumstances, may induce unrealistic appraisals, fostering superficial information processing, and reducing motivation to pursue challenging goals (Forgas and East, 2008; Pekrun and Perry, 2014). For language learners, unrealistic optimism may lead to an overestimated sense of control over the learning tasks and situation, hope for an easy shortcut to achievement, and less systematic planning and preparation, which will diminish their chances of success (Komorowska, 2016).

## A Theoretical Model of Second Language Emotions and Positive Psychology

**Figure 1:**
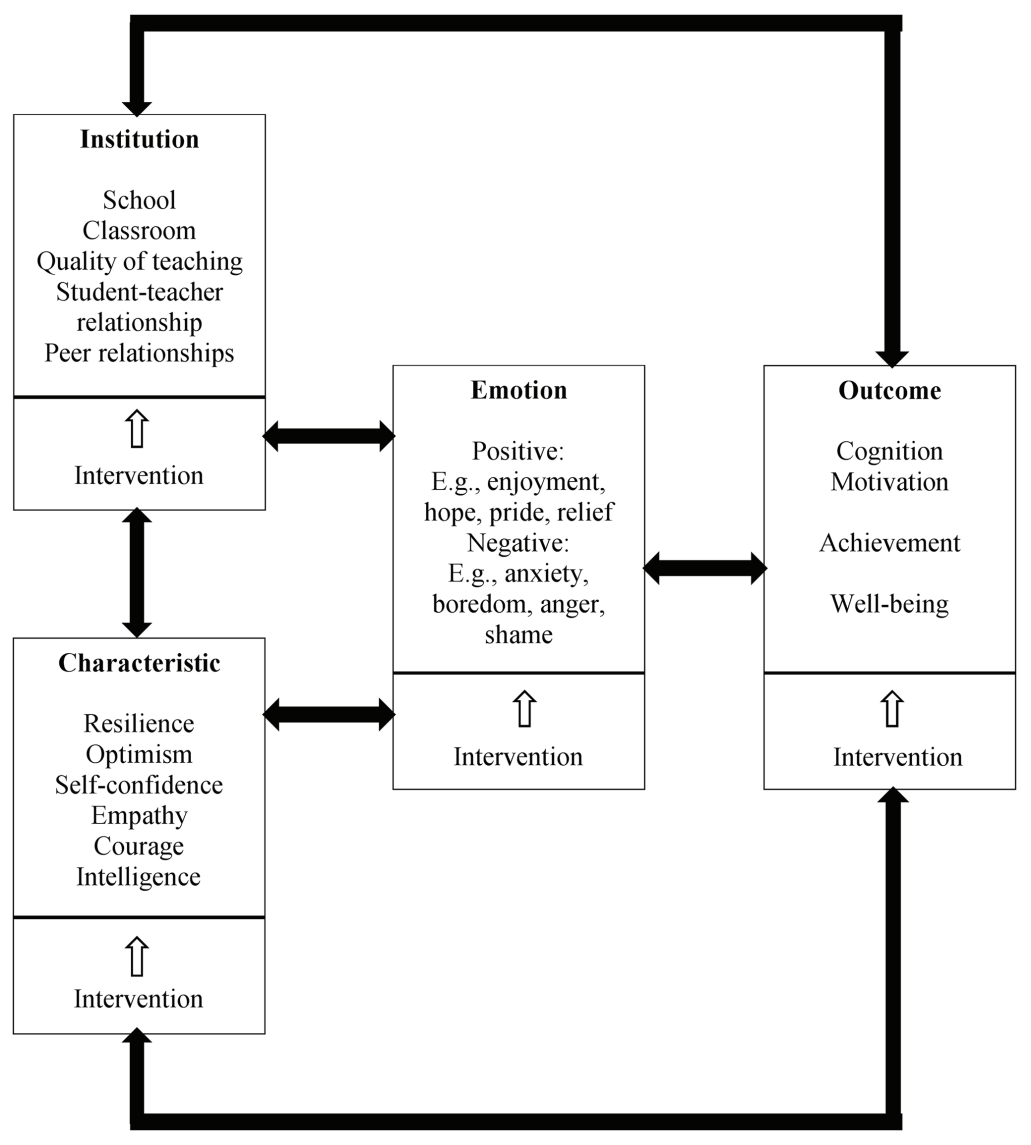
L2EPP (second language emotions and positive psychology) model integrating three pillars of PP with antecedents, outcomes, and interventions of L2 emotions.

Building on emotion theories and research from the fields of psychology, education, and SLA (e.g., Fredrickson, 2001; Pekrun, 2006; Dewaele et al., 2018; Shao et al., 2019), we now propose a second language emotions and positive psychology (L2EPP) model (see Figure 1) integrating the three core components of PP, that is, positive emotions, adaptive characteristics, and good institutions with the antecedents, outcomes, and interventions of L2 emotions. We begin by describing the theoretical framework before presenting empirical support for each aspect. As can be seen from Figure 1, characteristics and institutions represent individual and environmental antecedents of L2 emotions, respectively, and they are expected to be reciprocally linked with emotions and their learning outcomes in a feedback loop model. With emotion being placed at the center, each component of the model contains elements situating different aspects of PP into the L2 context and may exert both direct and indirect effects on the elements in other components. These elements may also interact with one another and with contextual factors (e.g., culture, policy, and language group, which are out of scope of the discussion) to co-determine language learners’ emotions, achievement, and well-being.

The theoretical assumptions underlying the L2EPP model are in line with both Pekrun’s (Pekrun, 2006; Pekrun and Perry, 2014) control-value theory of achievement emotions (CVT) and Fredrickson’s (Fredrickson, 2001; Fredrickson and Joiner, 2018) BBT. The CVT posits that students experience specific learning or achievement-related emotions when they feel in control, or out of control, of achievement activities and outcomes that are subjectively important, implying that perceived control and perceived value are proximal determinants of classroom emotions (Pekrun, 2006). Importantly, the CVT places emotion at the center of its framework (i.e., emotion is seen as both an independent and a dependent variable) and explicitly emphasizes the direct, indirect, and bidirectional effects as well as associated interventions among environmental features, appraisals, emotions, and achievement outcomes (Pekrun and Perry, 2014). On the other hand, the BBT postulates that experiences of positive emotions broaden people’s momentary thought-action repertoires and undo the lingering effects of negative emotions, which in turn serves to build their enduring personal resources, ranging from physical and intellectual resources to social and psychological resources (Fredrickson, 2001). The relationships among positive emotions, cognitive thinking, coping, and well-being are reciprocal, triggering an upward spiral (Fredrickson and Joiner, 2002).

The distinctions between these theories lie in the fact that the BBT is one of the founding blocks and major theories of PP, and it was introduced into the field of emotions and SLA as a fundamental theory for advocating the current PP movement in second language learning (MacIntyre and Gregersen, 2012; Dewaele and MacIntyre, 2014; Dewaele et al., 2019a). The CVT is based on contemporary appraisal theories of emotions (Scherer, 2013) and integrates various established theories in educational psychology (e.g., expectancy-value theory, transactional approaches of emotions, and attributional theory of motivation; see Pekrun et al., 2011). It was developed in general education aiming to expand the scope of emotion investigated beyond test anxiety in academic contexts as well as to systematically examine the antecedents and outcomes of students’ diverse achievement emotions (Pekrun et al., 2002). As a domain-general emotion theory, the CVT has only recently been applied to the domain of language learning (e.g., Piniel and Albert, 2018; Shao et al., 2019, 2020; Davari et al., 2020). While the BBT focuses on the effects of positive emotions on cognitive and psychological processes, the CVT attends to both positive and negative emotions together with their antecedents and outcomes.

In spite of the differences, these two theories share some common basic assumptions. Both CVT and BBT emphasize the crucial role of positive emotions (e.g., enjoyment, hope, pride, and contentment) in relation to cognitive thinking, personality traits, physical health, psychological well-being, and social environment as well as the reciprocal relationships among these variables. The value of cultivating positive emotions and fine-tuning negative emotions for the promotion of intellectual growth and psychological functioning is also prominent in both. The two theories are complementary for building our L2EPP model, which is designed for putting forward a series of testable hypotheses for L2 researchers and practitioners who are willing to devote their time and efforts into the exciting research area of PP and emotions in SLA.

Specifically, as delineated in the L2EPP framework (see Figure 1), we propose that academic environments/institutions can shape students’ learning characteristics, which then go on to influence their emotions toward language tasks. For example, a school/classroom climate featuring positive teacher and peer relationships can foster students’ positive characteristics such as self-confidence and optimism, which are likely to generate positive emotions such as enjoyment of learning, hope, and pride. These emotions can in turn affect the cognitive and motivational processes involved in language learning and subsequent achievement, which ultimately influences psychological well-being. Reciprocally, students’ psychosomatic health can also influence their language achievement, cognition, and motivation, which may have differential effects on their language learning emotions, for example, academic success might strengthen positive emotions and failure would induce negative emotions (e.g., anxiety, shame, and boredom). Different emotions can then influence students’ learning characteristics such as curiosity and perseverance, which will impact features of the learning environment, such as teachers’ design and selection of language tasks and materials. All of these effects are bidirectional, triggering a feedback loop. L2 emotion interventions that focus on any dimension of the model are likely to bring syntonic effects to all other parts of the L2EPP model. It is noted that the aspects included in each component of the model are not exhaustive; they are examples, albeit the most pertinent ones based on past research and theory. Not only could L2 researchers systematically study particular aspects of each component and how they relate to one another through possible mediation/moderation mechanisms, but also exploratory research could be conducted to identify other elements of each component that are not mentioned in the model.

The L2EPP model intends to guide L2 researchers to conduct innovative research incorporating PP into the particular area of emotions and SLA, as well as to help language teachers design PP interventions targeting students’ affective experiences in the classroom. In the following paragraphs, we illustrate each component of this model by merging recent emotion research in language and education with the preliminary applications of PP in SLA. Then, we discuss potential PP interventions, which may be applied to the field of L2 emotions based on each component. Finally, we suggest directions for future research and implications for language teaching based on each dimension of the model.

### Empirical Findings

#### Institutions

According to Peterson (2006, p. 20), “positive institutions can promote the development and display of positive characteristics, which in turn facilitate positive subjective experiences.” From a PP perspective, school is a unique institution that plays a role not only in fostering language learning, but also in the personal development and affective growth of teachers and students (Gabryś-Barker, 2016). However, institutional factors are the least investigated among the three pillars of PP by L2 emotion researchers (MacIntyre, 2016). Possible reasons for this situation include the multiple determinants of school environment, difficulty in assessing these factors, and different perceptions of these features by teachers and students (Gabryś-Barker, 2016). As Dörnyei (2014, p. 46) stated, “the complex character of school climate calls for a multidisciplinary approach which makes use of research findings from motivational psychology, educational studies and second language research.”

In general education, the CVT proposes that institutional factors (e.g., school, classroom, and teaching quality; see Figure 1) are distal antecedents of achievement emotions, which can have a collective influence on both teachers’ and learners’ emotions, resulting in a change of emotional experience for everyone in that particular environment (Pekrun, 2006). Supporting this proposition, research has shown that a classroom environment conveying positive relationships, enjoyment, and mastery goal orientation positively predicts students’ academic enjoyment, engagement, and achievement and negatively predicts their anxiety at both classroom and individual levels (Arens et al., 2015; Frenzel et al., 2018).

Specifically, Frenzel et al. (2018) examined the relationship between teacher and student enjoyment in math classes. Findings showed that teacher and student enjoyment were positively related even when controlling for students’ previous mathematics enjoyment, and that the effect of teacher enjoyment on student enjoyment was mediated by teacher enthusiasm. Lazarides and Buchholz (2019) investigated the relation between students’ perceived teaching quality in math classrooms and enjoyment, anxiety, and boredom among 6,020 German middle school students. Multilevel regression analyses showed that teacher support and classroom management were negatively related to student-level anxiety and boredom. Teacher support was positively related to enjoyment and negatively related to anxiety at the classroom level. Classroom management was negatively related to classroom-level boredom. Recently, employing doubly-latent multilevel analysis, Khajavy et al. (2018) examined the influence of classroom environment on students’ emotions in second language learning from the PP perspective. Results showed that a positive classroom climate featuring by teacher support and student cohesiveness positively predicts students’ enjoyment, but negatively predicts their anxiety. Enjoyment and anxiety also mediated the relationship between classroom environment and willingness to communicate at the classroom level.

#### Characteristics

The BBT (Fredrickson, 2001) suggests that positive characteristics such as resilience, perseverance, and optimism can help people undo the protracted effects of negative emotions quickly and experience more positive emotions that improve their cognitive functioning and psychological well-being. In the same vein, the CVT (Pekrun, 2006) proposes that personality traits like emotional intelligence and self-confidence related appraisals are more immediate antecedents of achievement emotions that can have a significant impact on learners’ emotional experiences. Educational research studies conducted so far have supported these propositions.

For example, using the BBT, Seaton and Beaumont (2015) examined the relationships between students’ positive emotions (awe and amusement), resilience, personal goals, and subjective well-being. Results revealed that positive emotions predicted increased resilience and more personal growth goals. Resilience partially mediated the relationship between positive emotions and well-being. Moreover, following the CVT, Heckel and Ringeisen (2019) investigated the proposed structural relationships between self-efficacy, cognitive appraisals, achievement emotions, and learning outcomes among 220 university students in an online learning environment. Findings showed self-efficacy was positively related to perceived control, interests, and pride, but was negatively correlated with anxiety. Control and interest mediated the relationships between self-efficacy and satisfaction/competence gain, respectively, while pride mediated the relationship between self-efficacy and competence gain.

In SLA, adapting the PP perspective, Wei et al. (2019) investigated the effect of grit on FLE and foreign language performance (FLP) among 832 middle school students in China. Results indicated that grit positively affected FLE and FLP, and the relationship between grit and FLP was mediated by FLE. Positive classroom environment also moderated the relationship between grit and FLE, and between grit and FLP. Similarly, Teimouri et al. (2020) examined the relationship between grit, as measured by consistency of interest and perseverance of effort, with emotion, motivation, and language performance. Findings showed that FL grit positively correlated with enjoyment, motivational variables (intended effort, willingness to communicate, and attention), and a series of language achievement measures (grammar, speaking, GPA, and self-rating), but negative correlated with anxiety. In another study, Li (2020) investigated the impact of trait emotional intelligence on Chinese students’ FLE and achievement from the perspective of PP. Results demonstrated that emotional intelligence is a positive predictor of language enjoyment and achievement. FLE partially mediated the relationship between emotional intelligence and self-reported/actual language performance.

#### Emotions

As previously mentioned, from a PP perspective, current L2 emotion research needs to expand the scope of both positive and negative emotions investigated. In the fields of psychology and education, Pekrun and colleagues’ research has demonstrated that there are qualitative and quantitative differences (e.g., content, intensity, valence, activation, and object focus) between discrete positive emotions and negative emotions (Frenzel et al., 2007; Goetz et al., 2010; Pekrun et al., 2011). According to the CVT (Pekrun, 2006), pleasant achievement emotions (e.g., enjoyment, hope, and pride) are posited to be jointly caused by high perceived control and high positive value, whereas unpleasant achievement emotions (e.g., anger, anxiety, shame, hopelessness, and boredom) are assumed to be elicited as a joint function of perceived lack of control and high negative value. Moreover, these discrete positive emotions and negative emotions are presumed to differentially relate to a wide range of learning outcomes (e.g., motivation, cognition, interest, learning strategies, and performance; see Pekrun et al., 2011). Although this line of emotion research has traditionally tended to focus on domain-general emotion variables, such as general test anxiety, or on students’ math-related emotions (e.g., Goetz et al., 2010; Pekrun et al., 2017), the construct of achievement emotions has recently attracted increasing attention from L2 researchers (e.g., Starkey-Perret et al., 2018; Shao et al., 2019, 2020; Davari et al., 2020). As Davari et al. (2020, p. 4) pointed out, “Adherence to a complex system of learning emotions would set L2 researchers free of a narrow, discrete approach to investigating language emotions. This complex system would also protect the SLA field from the criticism levelled against the perspectives on learning emotions that would draw on PP (e.g., dichotomization of positive emotions versus negative emotions and measurement problem).”

Specifically, employing a short-version of the achievement emotion questionnaire (AEQ; Pekrun et al., 2005, 2011), Starkey-Perret et al. (2018) examined the impact of two FL teaching approaches (i.e., traditional approach vs. task-based teaching) on learners’ two positive emotions (enjoyment and pride) and five negative emotions (anger, anxiety, shame, hopelessness, and boredom) in an urban middle school in France in three settings (class, learning, and test). Results of the study provided support for the reliability and validity of the AEQ for measuring language learning emotions. Similarly, Davari et al. (2020) investigated the psychometric properties of the class-related AEQ among 784 Iranian EFL learners. Exploratory factor analyses and confirmatory factor analysis (CFA) substantiated the internal structure of the AEQ for measuring eight emotions (enjoyment, hope, pride, anger, anxiety, shame, hopelessness, and boredom) in language learning. Multi-group CFA also demonstrated the measurement invariance of the AEQ across gender and learning contexts (school vs. institution). Very recently, Shao et al. (2020) examined the effects of perceived control and value on language emotions and performance as well as the moderated mediation effects of appraisals on achievement through emotions among Chinese college EFL students (*N* = 550). Results indicated that students experienced more positive emotions (enjoyment, hope, and pride) and less negative emotions (anger, anxiety, shame, hopelessness, and boredom) and achieved better language performance when they felt confident about language learning and found the learning activities and outcomes important and interesting. The interactive effects of control and value appraisals on FL performance were also mediated by enjoyment, hope, pride, and hopelessness.

#### Outcomes

Achievement and well-being are the ultimate goals of fostering learners’ adaptive emotions in language classrooms. This is in line with the goal of “positive education,” which explicitly aims to combine academic goals with the promotion of well-being for learners (Seligman et al., 2009). It is well-documented in psychology and education that the effects of emotions on learners’ achievement and psychological well-being depend on the interplay between various cognitive and motivational mechanisms (Pekrun et al., 2011; Fredrickson and Joiner, 2018; see above). According to the BBT and CVT, positive emotions can facilitate holistic thinking and creative problem solving, broaden the scope of attention and cognition, promote mastery approach goal, and enhance intrinsic motivation and long-term efforts, which eventually lead to better performance and well-being (Fredrickson and Branigan, 2005; Pekrun et al., 2011). On the other hand, negative emotions may cause divided attention and reduced cognitive resources, but can promote analytical thinking, facilitate emotion congruent memory and retrieval, and stimulate extrinsic motivation to invest effort (Pekrun and Perry, 2014). Thus, negative emotions can have variable effects on learning and well-being (e.g., López and Cárdenas, 2014), although negative consequences on overall academic performance likely outweigh any beneficial effects for most students (e.g., Pekrun et al., 2017).

However, existing L2 research has almost exclusively focused on the effects of emotions on language performance, leaving other language outcomes (such as cognition, motivation, and well-being) understudied. Among the few studies that have examined relations between emotions and motivation, most of them centered around Gardner’s (1985) social-educational model and Dörnyei’s (2005) L2 motivational self-system. From the PP perspective, supporters of the two theories argued that learners’ positive attitudes and behaviors (e.g., willingness to communicate) toward L2 learning or determination to deal with the discrepancy between their present self and their ideal L2 self would all require a strong sense of enjoyment for acquiring the target language (Teimouri, 2017; Dewaele et al., 2018; MacIntyre et al., 2019). Preliminary research findings supported this claim showing that L2 motivation and performance were positively related to enjoyment, but negatively correlated with anxiety (e.g., Chow et al., 2018; Saito et al., 2018).

Recently, pioneering L2 researchers have started to adapt the construct of achievement emotion to SLA and found similar motivational effects in a broader scope of emotions. For instance, Méndez-Aguado et al. (2020) examined the influence of emotions on motivation and performance among 394 students who learned French as a foreign language. Findings showed that positive emotions (enjoyment, calmness, and pride) positively predicted motivation, while negative emotions (anxiety, hopelessness, boredom, and embarrassment) negatively predicted it. Motivation toward French learning positively related to leisure habit to learn French and academic performance. Shao et al. (submitted manuscript) examined the relations between eight discrete emotions, motivational variables, and language performance in a sample of 1,021 Chinese EFL students. Results show that students’ enjoyment, hope, and pride were positively correlated with intrinsic motivation, extrinsic motivation, self-regulation, and FL performance; while the opposite trend was generally found among anger, anxiety, shame, hopelessness, and boredom.

### PP Interventions and L2 Emotions

Peterson (2006, p. 25) advocated that PP is “not a spectator sport”, that the field has taken on a mandate to develop practical interventions that help to create personal growth. Emotion-centered language activities targeting any part of the L2EPP model may empower positive transformation and growth among learners through the acquisition of essential life skills, thereby generating beneficial impact on the individual and their surroundings via emotion contagion (Hatfield et al., 1994). L2 educators may avail themselves of PP interventions tailored to different aspects of the L2EPP model for promoting students’ emotional well-being and linguistic competence. Some pioneering studies already exist for each dimension of the L2EPP, which we exemplify below.

Regarding interventions focused on the institutional component, Gabryś-Barker (2016) explored the possibility of applying PP activities to enhance emotional climate in language classrooms. Results show that EFL teachers’ awareness of the indicators of positive classroom atmosphere, understanding of teachers’ and students’ contribution to it and ability to use PP knowledge and techniques to adjust teaching are positively linked with both teachers’ and students’ affective well-being. Useful tips include creating a form of mutual responsibility for cultivating a positive learning environment and relationship between teachers and students; supporting students’ emotional needs; and engaging students in activities such as experience sharing, small talks, and collaborative assignments, which can boost connectedness and group cohesion.

Turning to positive characteristics interventions, Piasecka (2016) investigated whether working on poetry with PP activities can support learners’ character strengths. The findings show that this training can help learners develop language proficiency, imagination, and sensitivity and promote adaptive characteristics such as creativity, open-mindedness, courage, social intelligence, and appreciation of beauty. These enhanced characteristics show signs of helping students move toward their possible ideal L2 self. Students also reported increased self-efficacy, satisfaction, and happiness after the course. Moreover, adopting an emotional intelligence (EI) intervention program, Li and Xu (2019) examined the extent to which EFL learners’ FLE and FLA were linked to trait EI. The results showed that the EI intervention was effective in increasing EFL learners’ EI, boosting their FLE and reducing their FLA.

Concerning direct L2 emotion interventions, Kossakowska-Pisarek (2016) incorporated vocabulary training strategies with facets of self-control such as commitment control, metacognitive control, satiation control, emotion control, and environmental control to probe their influences on learners’ emotion, motivation, and performance. Learners were asked to set up their own goals, analyze them, implement strategies and monitor the process that would help them negotiate emotional responses to achieving or not achieving their goals. Findings showed that these activities can potentially raise students’ emotion awareness, enhance their commitment and motivation, and promote their positive emotions and language performance.

Finally, as reflected in the L2EPP model, any interventions focused on language outcomes would likely influence their antecedents and vice versa. For instance, applying six PP interventions (gratitude, altruism, music, pets, exercise, and laughter) into the language context, Gregersen (2016) paired experienced trainees of the PP approach with L2 learners to examine the efficacy of these procedures. Qualitative and quantitative findings showed that these activities were able to elevate students’ mood and positive emotions, and students reported greater feelings of engagement and well-being. Similarly, Mercer et al. (2018) enlisted the promotion of positive language education among L2 learners by using principles such as content and language integrated learning, which can have the potential to improve linguistic competence and subjective well-being at both individual and institutional levels.

It should be noted, however, that these interventions are still in the trial period and some of the criticisms of PP mentioned by Lazarus (2003), such as over-reliance on cross-sectional data, inadequate attention to within-between group differences, and poor quality of measurement, remained unsolved in these studies. Thus, language teachers are advised to critically examine empirical support for the interventions, understand how these interventions are supposed to function, and evaluate their outcomes before putting them into large-scale practice. L2 researchers may collect longitudinal data (Elahi Shirvan and Taherian, 2018), employ multilevel analysis (Khajavy et al., 2018), and adapt established and validated instruments from psychology and education (Seligman et al., 2009; Davari et al., 2020) when testing the effectiveness of emotion interventions that target any dimension of the L2EPP model in the future.

### Research Suggestions and Pedagogical Implications

The aforementioned empirical studies have demonstrated the theoretical utility of the L2EPP model for merging emotion research in language and education with PP concepts. However, this line of research is still in its infancy, and there is ample room for exploring each dimension of the model in the future. For example, previous research to date has only tapped relations between teacher and student emotions (Frenzel et al., 2018), leaving the influence of peer emotions largely neglected. Students spend a larger amount of time in the company of peers in their school life and the need for students to communicate and interact with one another is greater in language classrooms than for other subjects due to the communicative demands of language acquisition (Shao et al., 2019). Thus, language researchers may consider investigating how students’ language learning emotions are influenced by the emotional experiences of their peers through processes such as emotional contagion (Hatfield et al., 1994) or social appraisals (Parkinson and Manstead, 2015). By implication, this may also suggest that language teachers can promote students’ adaptive emotions by nurturing positive peer relations. One way to achieve this is through encouraging collaborative learning in and out of the classroom, which may help with developing positive interdependence and a supportive learning atmosphere among students (Johnson and Johnson, 2009).

L2 researchers may also consider expanding the scope of positive characteristics investigated to include, for instance, courage, humor, resolution, open-mindedness, altruism, curiosity, and empathy. For example, empathy, as an important element in Oxford’s (2016) EMPATHICS model, may play a vital role in shaping language learning emotions given that it relates to learners’ competence to understand the minds and emotions of interlocutors and develop positive relationships with them during intercultural communication, social interaction, and language use (Mercer, 2016). L2 researchers can investigate the influence of empathy on a wide range of positive and negative emotions (e.g., enjoyment, hope, pride, anger, shame, and boredom) as well as the potential mediating role of empathy in the relationship between institutional factors and language learning emotions. Pedagogically, language teachers can employ various intervention tools (e.g., role-play, movies, drama, reflective journal, and reading literature) to help learners foster their empathy (Mercer, 2016). One effective method can be through the use of role-play, by using either real or imaginative characters. Acting helps language learners to gain deeper insight into the minds and emotions of others. This vicarious experience enables them to be more considerate and resourceful in handling the relationship with peers and teachers as well as their own language learning emotions (Shao et al., 2012).

As for the focal area of L2 emotions, researchers may examine discrete positive and negative emotions in language learning in a more nuanced manner. In line with the two-dimension circumplex model of affect in psychology (Linnenbrink, 2007), Pekrun et al. (2002) has classified achievement emotions in educational settings based on valence and activation. Accordingly, positive activating emotions (hope, pride, and enjoyment), negative deactivating emotions (boredom and hopelessness), negative activating emotions (anger, anxiety, and shame) and positive deactivating emotions (relief) are presumed to have distinguished influence on motivation, self-regulation, effort, and performance (see Pekrun, 2006; Pekrun et al., 2011). L2 emotion researchers may probe how multiple types of emotions differentially link with their antecedents and language outcomes as well as the potential mediation effects of emotions between institutional factors, positive characteristics, and language achievement. Language educators may also heed to the unique roles of different types of emotions experienced by students. It is advised that teachers can help students capitalize on the double-edged sword of both discrete positive emotions and negative emotions and maintain the equilibrium of their effects on language learning. Intervention programs specifically targeting students’ achievement emotions such as attributional retraining (Hall et al., 2016) and value induction (Harackiewicz and Priniski, 2018) have already become available for language teachers.

Regarding language outcomes of emotions, one of the foremost areas might be to consider how emotions relate to well-being in language learning and teaching. Well-being is an aim, outcome, and contributor to the language learning process and can facilitate personal growth and social transformation (MacIntyre et al., 2019). Language researchers may pay more attention to the function of emotions for physiological and psychological health and investigate the influence of discrete positive emotions and negative emotions on psychosomatic indexes such as heart rate, adrenaline, blood pressure, satisfaction, mental health, and well-being (Gregersen et al., 2014). Possible mediation and moderation processes as well as the reciprocal links among institutional factors, learner characteristics, emotions, cognition, motivation, and well-being might also be probed (Fredrickson, 2001; Pekrun, 2006). In line with the goal of positive language education, L2 educators may explicitly aim to combine the acquisition of linguistic skills with the promotion of well-being in the classroom and school (Mercer et al., 2018). Language teachers can adopt a number of innovative instructional designs (e.g., participating in a fictional talk show or live interview; see Shao et al., 2012) to promote students’ positive emotions, language proficiency, and well-being. For example, teachers may select different types of background music to match teaching content, students’ preferences and learning contexts. The fine-tuned music may not only foster positive characteristics and adaptive emotions, but also facilitate language learning and psychological development (Kang and Williamson, 2014).

## Conclusion

The emergence of PP in SLA and the particular area of emotion and language learning resonate with a change of mentality among L2 researchers, shifting from a narrow, negative focus to a more positive, balanced approach for investigating language teaching and learning. PP is deemed to be an exciting topic to incorporate into the study of emotions and SLA because it encompasses facets like positive institutions, positive characteristics, and positive emotions, which are of primary relevance to the examination and cultivation of beneficial affective experiences in language classrooms. The initial research in this promising field is already producing new ideas, knowledge and insights for L2 emotion theorists (MacIntyre et al., 2016; Oxford, 2016) and proposing practical interventions for language teachers (Gregersen, 2016; Dewaele et al., 2018). In this article, we propose an L2EPP model that enables us to have a more holistic and systematic view on the issue of emotions and language learning from the lens of PP, recognizing the importance of institutional factors and character strengths, together with effective intervention designs in shaping language learners’ emotional well-being and achievement. By aligning the BBT (Fredrickson, 2001) with the CVT (Pekrun, 2006), the L2EPP model has the potential to provide a framework of language emotions that would effectively address criticism against the PP approach in SLA. The advent of PP into the area of emotions and language learning broadens the horizon for applied linguists and language practitioners, and in this research endeavor, we also offer new input from our unique experiences and perspectives to enrich its theories and practice, which can lead to cross-fertilization of new ideas. In building the L2EPP model with its many hypotheses, mechanisms, and practices yet to be tested, we envision a bright future for its utility in the area of emotions and SLA.

## Data Availability Statement

The original contributions presented in the study are included in the article/supplementary material, and further inquiries can be directed to the corresponding author.

## Author Contributions

KS guided and drafted the review. LN co-wrote and designed the article. GK co-wrote and edited the review. FL reviewed and revised the manuscript. All authors contributed to the article and approved the submitted version.

### Conflict of Interest

The authors declare that the research was conducted in the absence of any commercial or financial relationships that could be construed as a potential conflict of interest.
