# Corrigendum: Emotions and Instructed Language Learning: Proposing a Second Language Emotions and Positive Psychology Model

**DOI:** 10.3389/fpsyg.2020.605188

**Published:** 2020-11-26

**Authors:** Kaiqi Shao, Laura J. Nicholson, Gulsah Kutuk, Fei Lei

**Affiliations:** ^1^School of Foreign Languages, Hangzhou Dianzi University, Hangzhou, China; ^2^Faculty of Education, Edge Hill University, Ormskirk, United Kingdom; ^3^Faculty of Education, Edge Hill University, Ormskirk, United Kingdom; ^4^School of Foreign Studies, Center for Language Cognition and Assessment, South China Normal University, Guangdong, China

**Keywords:** emotion, second language acquisition, positive psychology, theory, learning, teaching

**Kaiqi Shao** should not have been included as a corresponding author in the published article. **Fei Lei** was the sole corresponding author of this article.

The authors apologize for this error and state that this does not change the scientific conclusions of the article in any way. The original article has been updated.

